# Supplementary material for: A UMLS-based spell checker for natural language processing in vaccine safety
Source: BMC Med Inform Decis Mak. 2007 Feb 12;7:3. doi: 10.1186/1472-6947-7-3 (PMC1805499; doi:10.1186/1472-6947-7-3)
Supplement: Additional file 13 — RAP application directory. Sets up directory for RAP (RDF application for PHP) source code [file 1472-6947-7-3-S13.gz › rap/doc/phpdoc/__filesource/fsource_default__vocabularyRDF_RES.php.html]

xml version="1.0" encoding="iso-8859-1"?


File Source for RDF\_RES.php


# Source for file RDF\_RES.php

Documentation is available at RDF\_RES.php

```
1. <?PHP
2. // ----------------------------------------------------------------------------------
3. // Resource Description Framework (RDF) Vocabulary (ResResource)
4. // ----------------------------------------------------------------------------------
5. // Version                   : 0.9
6. // Authors                   : Daniel Westphal (dawe@gmx.de)
7. //
8. // Description               : Wrapper, defining resources for all terms of the
9. //                               Resource Description Framework (RDF).
10. //                               For details about RDF see: http://www.w3.org/RDF/.
11. //                                Using the wrapper allows you to define all aspects of
12. //                             the vocabulary in one spot, simplifing implementation and
13. //                             maintainence.
14. //
15. // ----------------------------------------------------------------------------------
16. // History:
17. // 02-21-2003                 : Initial version
18. // ----------------------------------------------------------------------------------


22. class RDF_RES{

24. // RDF concepts (constants are defined in constants.php)

26. function ALT()
27. {
28. return  new ResResource(RDF_NAMESPACE_URI . RDF_ALT);

30. }

32. function BAG()
33. {
34. return  new ResResource(RDF_NAMESPACE_URI . RDF_BAG);

36. }

38. function PROPERTY()
39. {
40. return  new ResResource(RDF_NAMESPACE_URI . RDF_PROPERTY);

42. }

44. function SEQ()
45. {
46. return  new ResResource(RDF_NAMESPACE_URI . RDF_SEQ);

48. }

50. function STATEMENT()
51. {
52. return  new ResResource(RDF_NAMESPACE_URI . RDF_STATEMENT);

54. }

56. function RDF_LIST()
57. {
58. return  new ResResource(RDF_NAMESPACE_URI . RDF_LIST);

60. }

62. function NIL()
63. {
64. return  new ResResource(RDF_NAMESPACE_URI . RDF_NIL);

66. }

68. function TYPE()
69. {
70. return  new ResResource(RDF_NAMESPACE_URI . RDF_TYPE);

72. }

74. function REST()
75. {
76. return  new ResResource(RDF_NAMESPACE_URI . RDF_REST);

78. }

80. function FIRST()
81. {
82. return  new ResResource(RDF_NAMESPACE_URI . RDF_FIRST);

84. }

86. function SUBJECT()
87. {
88. return  new ResResource(RDF_NAMESPACE_URI . RDF_SUBJECT);

90. }

92. function PREDICATE()
93. {
94. return  new ResResource(RDF_NAMESPACE_URI . RDF_PREDICATE);

96. }

98. function OBJECT()
99. {
100. return  new ResResource(RDF_NAMESPACE_URI . RDF_OBJECT);

102. }

104. function DESCRIPTION()
105. {
106. return  new ResResource(RDF_NAMESPACE_URI . RDF_DESCRIPTION);

108. }

110. function ID()
111. {
112. return  new ResResource(RDF_NAMESPACE_URI . RDF_ID);

114. }

116. function ABOUT()
117. {
118. return  new ResResource(RDF_NAMESPACE_URI . RDF_ABOUT);

120. }

122. function ABOUT_EACH()
123. {
124. return  new ResResource(RDF_NAMESPACE_URI . RDF_ABOUT_EACH);

126. }

128. function ABOUT_EACH_PREFIX()
129. {
130. return  new ResResource(RDF_NAMESPACE_URI . RDF_ABOUT_EACH_PREFIX);

132. }

134. function BAG_ID()
135. {
136. return  new ResResource(RDF_NAMESPACE_URI . RDF_BAG_ID);

138. }

140. function RESOURCE()
141. {
142. return  new ResResource(RDF_NAMESPACE_URI . RDF_RESOURCE);

144. }

146. function PARSE_TYPE()
147. {
148. return  new ResResource(RDF_NAMESPACE_URI . RDF_PARSE_TYPE);

150. }

152. function LITERAL()
153. {
154. return  new ResResource(RDF_NAMESPACE_URI . RDF_PARSE_TYPE_LITERAL);

156. }

158. function PARSE_TYPE_RESOURCE()
159. {
160. return  new ResResource(RDF_NAMESPACE_URI . RDF_PARSE_TYPE_RESOURCE);

162. }

164. function LI()
165. {
166. return  new ResResource(RDF_NAMESPACE_URI . RDF_LI);

168. }

170. function NODE_ID()
171. {
172. return  new ResResource(RDF_NAMESPACE_URI . RDF_NODEID);

174. }

176. function DATATYPE()
177. {
178. return  new ResResource(RDF_NAMESPACE_URI . RDF_DATATYPE);

180. }

182. function SEE_ALSO()
183. {
184. return  new ResResource(RDF_NAMESPACE_URI . RDF_SEEALSO);
185. }
186. }


189. ?>
```

Documentation generated on Fri, 17 Dec 2004 16:17:23 +0100 by phpDocumentor 1.3.0RC3
